# Cybersecurity in Healthcare: A Systematic Review and Narrative Analysis

**DOI:** 10.1055/a-2865-4206

**Published:** 2026-05-13

**Authors:** Clemens S. Kruse, Diane Dolezel, Ramalingam Shanmugam

**Affiliations:** 1 12337 College of Health Sciences, The University of Texas at El PasoEl Paso, TexasUnited States; 2 Department of Health Informatics and Information Management 7174 Texas State UniversitySan Marcos, TexasUnited States; 3 School of Health Administration, Texas State university, San Marcos, Texas, United States

**Keywords:** clinical data management, information assurance, cybersecurity, internet of things, risk management, clinical information systems, specific types, patient records

## Abstract

**Background:**

Cybersecurity attacks in healthcare have increased in number and severity over the last decade. Healthcare targets are ten times more valuable than financial targets because of the potential for fraudulent medical claims. Medical providers and administrators must ensure their IT professionals constantly scan the internal and external environment for techniques to prevent, detect, respond to, and report cyberattacks. This review provides a scan of the literature.

**Objective:**

This study aimed to review the literature for over 10 years for techniques for prevention, detection, response, and reporting of cyberattacks. An extended objective was to collect the effect on patient care that has been documented from cyberattacks.

**Methods:**

Following a published protocol and reporting standard, this systematic literature review queried four research databases for published works that described cybersecurity in healthcare. Grey literature was included.

**Results:**

Twenty-two articles provided 139 observations and 13 themes that described current techniques to secure the healthcare infrastructure. These articles stressed the importance of a trained workforce and a cyber-aware culture.

**Conclusion:**

IT professionals must adopt techniques that form proper organizational cyber resiliency and augment security through monitoring tools, standard IT maintenance and practice, encryption and data loss prevention, risk-based management, Artificial Intelligence, Explainable AI, active AI, network segmentation, governance and leadership, disabling legacy protocols and systems that cannot be updated, postevent analysis, and big data.

## Background and Significance

### Rationale

While cybersecurity can fit into a range of concepts, the few organizations that have underestimated its importance have done so at their peril. As this article will explain, cybersecurity attacks in healthcare are an increasing problem because healthcare targets have a 10-fold value on the dark web over identity theft. Due to its value, healthcare cyber-threats can afford to attract strong talent, so threats are constantly evolving. Cybersecurity resilience can fortify defenses enough to convince a threat actor to seek a softer target.

Generally, cybersecurity is “security relating to computer systems or the internet, especially that intended to protect against viruses or fraud.” 1 The America's Cyber Defense Agency expands on this definition as the “art of protecting networks, devices, and data from unauthorized access or criminal use and the practice of ensuring confidentiality, integrity, and availability of information.” 2 This definition is particularly relevant to healthcare because of the large volume of sensitive information handled by the industry. Since the release of the Institute of Medicine's book, “Crossing the Quality Chasm,” the healthcare industry has relied heavily on digital and web-enabled technology to enhance patient safety, efficiency, and effectiveness. 3 4 Unfortunately, this has also opened organizations to web vulnerabilities. The typical smart hospital contains infusion pumps, smart elevators, remote patient monitoring, smart Heating, Ventilation, and Air Conditioning (HVAC), and medication dispensing systems like Pyxis. Many of these assets are often connected to the network and integrated into the Internet of Medical Things (IoMT) or Internet of Health Things (IoHT), making them a target for cyberattacks. 4

The Health Information Management Systems Society lists many of the common stakeholders to proper cybersecurity: patients, workforce members, C-Suite, vendors/market suppliers. These stakeholders can be attacked through many avenues: phishing, email, and legacy systems. The physical security and network security can become breached, enabling payload packages like ransomware to be deployed. 5 6 The result can be realized in treatment delays, medical errors, or the leaking of patient data. 6 7 The risk to the organization is secondary: the primary concern is the threat to patients' privacy and public safety, particularly in the face of infectious disease response. 8 The cost of this risk was estimated at $10 trillion in 2023, but it is forecasted to increase to $24 trillion over the next 4 years. 8 These imperatives stress the importance of studying cybersecurity in healthcare.

In 2024, Hasegawa et al explored cybersecurity interventions to reduce vulnerabilities in low-to middle-income countries ( *n* = 20) in a systematic review. Researchers implemented the Essentials of Cybersecurity in Healthcare Organizations (ECHO). Fourteen countries were represented in this ECHO systematic literature review. Interventions were categorized by governance, context, organizational strategy, awareness, risk management, technical capabilities, training, and education. Their main conclusion was the lack of sufficient information available for study. Hasegawa et al recommended that organizations publish their experiences to enable others to respond appropriately. 7

Joshi explored informatics projects in healthcare to assess challenges, solutions, and future directions ( *n* = 25). He searched four research databases over 10 years. He identified four challenges to the implementation of informatics projects in healthcare: interoperability, financial constraints, data security, and user resistance. He suggested collaborative interoperability, innovative financing, enhanced cybersecurity, and a user-centered approach to address these challenges. 9

Salama et al explored cybersecurity in healthcare and its challenges ( *n* = 316). Researchers queried two research databases and identified 11 solutions for cybersecurity vulnerabilities in their scoping review. They highlighted six problem areas: poor security awareness in the organization, inadequate endpoint device management, insufficient continuity of operation plans, along with uncoordinated incident response, unbalanced spending (security vs. service delivery), and reckless remote working conditions. They also identified four primary attacks: malware, distributed denial of service (DDOS), ransomware, and phishing. 10

Dobrovolska et al used economic and mathematical modeling to analyze correlations between the Global Health Security Index (GHSI) and the Global Cybersecurity Index (GCSI) for 190 countries in 2021. They found strong correlations between legal measures and prevention, as well as technical measures and detection and reporting. This approach is interesting as it highlights the importance of augmenting technical safeguards with legal compliance to ensure prevention, detection, and reporting of security breaches. 11

### Objective

Researchers in this systematic literature review investigated the risks, vulnerabilities, and solutions within healthcare. Although the research primarily focused on implementation in the United States, international research studies were also scanned for risks, vulnerabilities, and solutions. The intent was to summarize the prevention, detection, response, recovery, and reporting techniques published in the literature and provide updated conclusions for clinicians, administrators, and IT professionals working in the healthcare IT space.

## Methods

### Eligibility Criteria

Researchers chose studies published in the last 10 years. This timeframe was used for two reasons: (1) the technology industry is fast-moving, and studies older than a decade may no longer be applicable, and (2) 10 years is a sufficient amount of time to see trends in the industry, as established by literature. 12 13 Researchers chose to eliminate systematic reviews from analysis to avoid a confounding effect. Both systematic and scoping reviews already summarize the literature. If researchers attempt to summarize the literature and include other publications that do the same, it risks overemphasizing certain security approaches or techniques.

### Information Sources

Researchers queried PubMed (MEDLINE), Science Direct, Web of Science (Scopus), and The Cumulative Index to Nursing and Allied Health Literature (CINAHL) for over 10 years using the following search string.

### Search Strategy

Reviewers created a Boolean search string combining key terms found in the literature and vetted using Medical Subject Headings (MeSH). The Boolean search string used was healthcare and (“computer security” or “internet of things” or “medical informatics”) and cyberattack. The same search string was used in all databases, but the filters used in each database slightly varied because the tools available were not the same.

### Selection Process

Because researchers followed a published protocol, search results were filtered and screened to ensure they were applicable to the purpose of the systematic review. 14 Ideally, the reviewers preferred to have randomized controlled trials or nonrandomized studies of interventions. However, because these are highly unusual in cybersecurity, the reviewers will capture all peer-reviewed and published works in the area.

### Data Collection Process and Data Items

Researchers used a standardized Excel spreadsheet as both a screening and data extraction tool. 14 This spreadsheet was standardized in the published protocol, and it included various fields like participants, intervention, results, outcomes, design, size, bias, effects, country of origin, statistics, security techniques, and quality assessments. Some of these fields were specific to this study, while others were required in the Preferred Reporting Items for Systematic Reviews and Meta-Analyses (PRISMA) standard ( PRISMA 2020 Checklist , available in the online version only). 15 The spreadsheet also assigned which abstracts to screen and analyze per author, which were about 20 and 10, respectively. A series of consensus meetings was used to ensure the researchers agreed on the purpose, approach, screening, and extraction. The lead author led these meetings.

### Study Risk and Reporting Bias Assessment

Researchers recognize the importance of analyzing only quality articles in order to make generalized inferences. Researchers chose the Johns Hopkins Nursing Evidence-Based Practice tool (JHNEBP) as an evaluation of quality. 16 This tool has two measures: Strength of Evidence, as defined by levels of study, and Quality of Evidence as defined by depth of evidence and clarity of study strategy. In addition to the quality assessment, researchers noted bias in each article read.

### Effect Measures

Where effects were reported, researchers noted each measure in a manner that enabled meta-analysis. While the Cohen *d* , Wald *w* , beta, and odds ratios were preferred, most of the material analyzed was categorized as grey literature. Mixed-methods studies were also included, where available.

### Synthesis Methods, Additional Analyses, and Certainty Assessment

After screening abstracts, researchers conducted a thematic analysis of the articles. 17 This technique involved reading each article multiple times and making notes during each reading. When observations were repeated across articles, they were noted as themes. The convergence of themes demonstrated the reliability of topics. Observations that stood alone were reported only as observations. Researchers used this technique as a form of sense-making. Researchers tabulated the themes and observations into affinity tables to show the frequency of their appearance. This was done not to imply importance, but only to report the data in a probabilistic manner. Researchers combined the narrative analysis with the quality assessment to perform a certainty assessment. A convergence of themes coupled with high-quality assessments of articles would enable a certainty assessment of high. Conversely, a lack of convergence and low-quality assessments would lead to a certainty assessment of low. The certainty assessment helped evaluate a level of confidence in the findings.

## Results

**Fig. 1:**
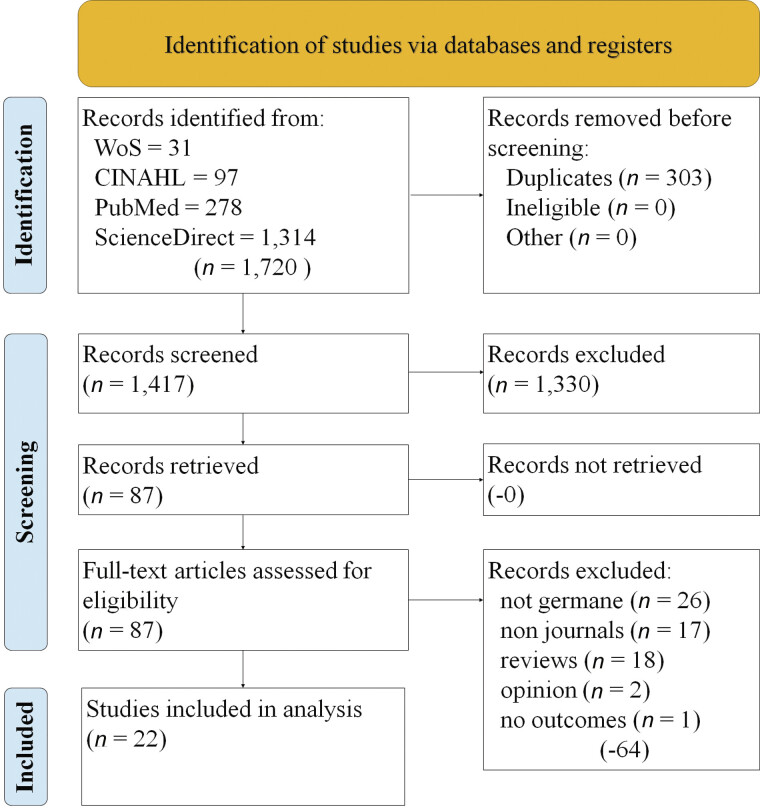
Article selection process.

The lead author assigned workload and led consensus meetings as planned. Each author screened about 20 abstracts and provided a narrative analysis of 12 full articles. This ensured each abstract and each article was processed by at least two researchers. Where there was conflict, the third author processed the work. A Cohen's kappa statistic was calculated to report the level of agreement between reviewers in the abstract screening process ( *k* = 0.84, strong agreement). 18 19 Statistically, agreement purely by chance was calculated to be 50%, but our level of agreement was calculated significantly higher. 18 Fig. 1 illustrates the article selection process to obtain the final group for analysis ( *n* = 25).

### Study Selection

Fig. 1 illustrates the article selection process. From the four research databases, abstracts were screened, and articles were selected for full analysis ( *n* = 22). Multiple reviewers agreed on abstracts for selection, and multiple reviewers simultaneously extracted data for analysis from these full articles. This practice fulfills the requirements of robustness in the AMSTAR2 (available in the online version only) standard. 20

### Study Characteristics

Reviewers followed the PRISMA checklist and extracted both standardized data fields and fields specific to the study.

Because most of the articles selected for analysis were white papers, most of the standard PICOS (participants, intervention, comparison, observation, and study design) were not reported. However, there were several studies that provided these details. The fields specific to the study were prevention, detection, response, recovery, and reporting techniques, effect on patient care, and hospital characteristics. Of the 22 studies in the group for analysis, six were from 2024 (27%), 21 22 23 24 25 26 four were from 2023 (18%), 27 28 29 30 four were from 2022 (18%), 31 32 33 34 3 were from 2021 (14%), 35 36 37 2 were from 2020 (9%), 38 39 1 was from 2019 (5%), 40 and 2 were from 2018 (9%). 41 42 No articles were selected from the years 2015 to 2017. Eleven of the 22 articles (50%) were from the Americas, 21 27 28 31 35 36 37 38 40 41 42 three of the 22 (14%) were from Europe, 25 29 32 six of the 22 (27%) were from Asia and the Middle East, 23 24 26 30 33 34 and the remainder were from Australia (2/22, 9%), 22 39 About 80% of the prevention, detection, reaction, recovery, and reporting techniques reported can be found in cyber resiliency models. Other techniques span risk-based management, artificial intelligence (AI), active AI, or Explainable AI (AIX), blockchain, or big data. Supplementary Table S1 (available in the online version only) provides the PICOS table as an additional explanation of study characteristics.

### Risk of Bias in Studies

Using the JHNEBP quality assessment tool, reviewers identified seven of 22 with strength-of-evidence of III (32%), while the remainder were strength-of-evidence IV (68%). Assessments of the quality of evidence were also made. Reviewers identified two of 22 (9%) studies of the highest quality (1), 13 of 22 (59%) of the middle quality (2), and only one of 22 (5%) of the lowest quality (3). According to JHNEBP's creators, a summary of multiple qualitative studies is considered high quality as long as it exercises a well-defined, reproducible search and appraisal strategy and finds congruence of results across studies. When we merge into high-quality opinions from subject-matter experts, we must check findings carefully to ensure they are accurate. The grey literature used was from professional magazines that publish works from subject-matter experts, and they employ professionals to fact-check underlying assumptions and their conclusions. In addition to the JHNEBP quality assessment tool, reviewers also made individual notes of bias within each study. The reviewers noted 22 instances of selection bias. Nineteen of the articles were written from the perspective of one country, while the other three were written from the perspectives of one region. The combination of the high-quality results from the quality assessment tool and consistency of results from the narrative analysis resulted in a certainty assessment of high.

Reviewers planned to use Cochran's Q to ensure the results were all below the rejection criteria. Unfortunately, only six of the 22 articles (27%) involved a study design beyond a white paper, which limited the scope of the statistic. 23 25 26 30 39 42 Quantitative synthesis/heterogeneity testing was not feasible due to limited and heterogeneous empirical evidence in combination with white papers.

### Results of Individual Studies

Table 1 tabulates the results of individual studies using themes from thematic analysis.

**Table 1:** Summary of analysis, sorted from most recent to oldest

Authors	Prevention, detection, response, and reporting	Prevention, detection, response, and reporting themes	Effects on patient care	Effects on patient care themes
James 21	Segmenting networks to reduce the amount of data or systems one person or system can access	Segment network	Disruption of workflows	Delay care
	Doctors could keep paper patient notes as a backup	Governance and leadership		
	Educating staff on passwords	Organizational cyber resiliency	Imaging can go missing	Missing care elements
	Multi-factor authentication	Access control	Lab results can go missing	Missing care elements
Kumar et al 22	Time-series classification models for identifying potential cyberattacks on the Internet of Health Things (IoHT)	Monitoring tools and technology	Not reported	
	Neighborhood component analysis to select inputs	AI, AIX, or active AI		
	Machine learning to identify cyberattacks in the IoHT network	AI, AIX, or active AI		
Abbou et al 23	Anti-malware software	Monitoring tools and technology	Surgeries rescheduled	Delay care
	Halt all elective surgeries and invasive procedures	Organizational cyber resiliency		
	Software-based response	Monitoring tools and technology	Patient transfers	Delay care
	Distribution of laptops with secured cellular Internet connection	Standard maintenance and practice	All nonlive-saving procedures rescheduled	Delay care
Aljuhani et al 24	Integrated intrusion detection	Monitoring tools and technology	Not reported	
	Efficient feature engineering	AI, AIX, or active AI		
	Comprehensive attack detection	Monitoring tools and technology		
	Explainability through Shapley additive explanation	AI, AIX, or active AI		
	Software-as-a-service (SaaS) Deployment on edge computing	Monitoring tools and technology		
	Machine learning to identify cyberattacks in the IoHT network	AI, AIX, or active AI		
Hore et al 25	Education and training	Organizational cyber resiliency	Not reported	
	Culture of awareness and personal responsibility	Organizational cyber resiliency		
	Cybersecurity practices are integrated with the normal workload	Organizational cyber resiliency		
	Update the IT infrastructure	Standard maintenance and practice		
Dhar and Majumder 26	Authentication, authorization, and access (AAA) control layer	Access control	Not reported	
	Elliptic Curve Cryptography (ECC)-based key management	Encryption and DLP		
	Elliptic Curve Diffie–Hellman Key Exchange	Encryption and DLP		
Dixit et al 27	Organizational cyber resiliency	Organizational cyber resiliency	Not reported	
	Governance and leadership	Governance and leadership		
	Risk-based management of cybersecurity	Risk-based management of cybersecurity		
	Cyber-risk management frameworks	Risk-based management of cybersecurity		
	Education and training	Organizational cyber resiliency		
	Monitoring tools and technology	Monitoring tools and technology		
Cartwright 28	Software patching	Standard maintenance and practice	Infusion pumps can be easily altered to change the rates of flow	Potential medical error
	Train hospital staff	Organizational cyber resiliency		
	Encryption	Encryption and DLP		
	Segment network	Segment network		
	Education and training	Organizational cyber resiliency		
Rehman et al 29	Alternate quantum random walks	Encryption and DLP	Not reported	
	Controlled Rubik's Cube transformations	Encryption and DLP		
	Integration of the Elliptic Curve Cryptosystem with Hill Cipher (ECCHC)	Encryption and DLP		
Moulahi et al 30	Blockchain-based federated learning	Blockchain	Not reported	
	Asymmetric key cryptography	Encryption and DLP		
	Hyperledger (Linux-based)	Blockchain		
	Real-time AI to interrogate packets	AI, AIX, or active AI		
	Protection at the physical, network (transport), and application layers	Organizational cyber resiliency		
	Machine learning to identify cyberattacks in the IoHT network	AI, AIX, or active AI		
Stokes 31	The incident was a ransomware attack resulting from an employee's phishing email on a work laptop that he took on vacation	Postevent analysis	Not reported	
	Creating a communication plan	Governance and leadership		
	COOP	Organizational cyber resiliency		
	Education and training	Organizational cyber resiliency		
	Encryption	Encryption and DLP		
	VPN	Standard maintenance and practice		
	Cyberattack plan	Organizational cyber resiliency		
	Regular policy and procedure review	Standard maintenance and practice		
Wells 32	education and training	Organizational cyber resiliency	Not reported	
Kumar et al 33	Blockchain secures EMR	Blockchain	Network traffic lags, which could lead to delays in care	Delay care
	Blockchain to secure EHR	Blockchain		
	Blockchain to secure remote patient monitoring	Blockchain		
	Blockchain to protect health insurance claims	Blockchain		
	Network segmentation	Segment network		
Kang and Kim 34	Proposed healthcare big data platform	Big Data	Not reported	
	Attribute-based encryption	Encryption and DLP		
	Encryption of immutable storage	Encryption and DLP		
	Blockchain	Blockchain		
	Access control through blockchain	Access control		
	Record real-time success or failure of user activities	AI, AIX, or active AI		
Relias 35	MFA	Access control	Not reported	
	Practice procedures	Organizational cyber resiliency		
	Establish procedures for security incidents	Organizational cyber resiliency		
	Disable legacy remote access protocols (RDP, SSH)	Disable legacy protocols and equipment that cannot be updated		
	Segment network	Segment network		
	Disconnect any system that cannot be updated	Standard maintenance and practice		
	COOP	Organizational cyber resiliency		
	Update and patch systems	Standard maintenance and practice		
	Third-party penetration testing	Organizational cyber resiliency		
	Encryption	Encryption and DLP		
	Regular backups	Organizational cyber resiliency		
	Test backups	Standard maintenance and practice		
	Store backups offsite	Standard maintenance and practice		
	Education and training	Organizational cyber resiliency		
	Forensic analysis of affected devices	Organizational cyber resiliency		
	Role-based security	Access control		
	Update firewalls	Monitoring tools and technology		
Relias 36	Secure IoT devices	Organizational cyber resiliency	Reverting to paper records delays care	Delay care
	Data loss prevention (DLP) solution	Encryption and DLP		
	Zero standing privilege environment	Access control		
	Intelligence gathering capabilities	Organizational cyber resiliency		
	Network segmentation	Segment network		
	Secure cloud-based apps	Standard maintenance and practice	Reverting to paper records delayed medication dispensing	Potential medical error
	Secure legacy systems	Disable legacy protocols and equipment that cannot be updated		
	Regular backups of immutable storage	Organizational cyber resiliency		
	Perform recovery ops if EHR is held for ransom	Organizational cyber resiliency		
	Real-time AI to interrogate packets	AI, AIX, or active AI		
Upendra 37	Prevention with passive network monitoring (PNM)	Organizational cyber resiliency	Anti-virus software can disrupt the functioning of IOMT	Potential medical error
	Use proactive network management to protect without disrupting the function of IOMT	Organizational cyber resiliency		
	Important to asset mgmt. to identify these systems using the NIST cybersecurity framework	Risk-based management of cybersecurity		
	Prevention: awareness and training	Organizational cyber resiliency		
	Data security	Monitoring tools and technology		
	Maintenance	Standard maintenance and practice		
	Overview of network security types	Risk-based management of cybersecurity		
	Medical device cybersecurity program	Organizational cyber resiliency		
	Identify programmatic gaps that signal the need for PNM	Risk-based management of cybersecurity		
	Anti-virus software	Monitoring tools and technology		
	Anti-malware software	Monitoring tools and technology		
	Access control	Access control		
	Host intrusion detection and prevention	Monitoring tools and technology		
	Management agents	Monitoring tools and technology		
	Application security	Organizational cyber resiliency		
	Behavioral analytics	Monitoring tools and technology		
	Distributed denial of service protection	Monitoring tools and technology		
	Email security	Monitoring tools and technology		
	Firewalls	Monitoring tools and technology		
	Mobile device security	Risk-based management of cybersecurity		
	Network segmentation	Segment network		
	Security information and event management	Organizational cyber resiliency		
	Web security	Monitoring tools and technology		
Kabir et al 38	Cybersecurity insurance that covers ransomware attacks and third-party contractors	Organizational cyber resiliency	Not reported	
	Before purchasing a policy, health care organizations should perform a comprehensive assessment of their information technology security capabilities and needs	Risk-based management of cybersecurity		
	Risk-based management assessment	Risk-based management of cybersecurity		
	Capabilities assessment	Organizational cyber resiliency		
	Education and training	Organizational cyber resiliency		
	Blockchain for medical records	Blockchain		
Dave et al 39	Analyze the impact of the cybersecurity event	Postevent analysis	Not reported	
	Establish multi-level decision-making	Organizational cyber resiliency		
	Establish strong horizontal and vertical leadership	Governance and Leadership		
	Establish proactive cybersecurity initiatives	Organizational cyber resiliency		
Ferrara 40	Education and training	Organizational cyber resiliency	Not reported	
Malmo 41	Training	Organizational cyber resiliency	Not reported	
	Multi-factor authentication	Access control		
	Segregate billing functions	Segment network		
	PT integrity in practice program preventing fraud, waste, and abuse	Standard maintenance and practice		
	A primer for physical therapists	Organizational cyber resiliency		
	A free guide to laws and regulations	Organizational cyber resiliency		
	Firewalls	Monitoring tools and technology		
	Anti-virus software	Monitoring tools and technology		
	Encryption	Encryption and DLP		
	Risk assessment	Risk-based management of cybersecurity		
	Ransomware response	Organizational cyber resiliency		
	system protection	Standard maintenance and practice		
Etges et al 42	Develop ERM programs (enterprise risk management)	Risk-based management of cybersecurity	Not reported	
	Risk-management culture	Risk-based management of cybersecurity		

Other observations collected in the data extraction process can be found in Appendix A (available in the online version only). Articles not chosen for this review, along with their reasons for rejection, can be found in Appendix B (available in the online version only). Depending on the Cyber Resiliency model chosen, reviewers could have categorized observations into only five themes: Cyber resiliency, Blockchain, Risk-based Management, Big Data, and a combination of AI, AIX, and active AI. However, reviewers chose to use additional themes because they provide valuable discussion on prevention, detection, response, recovery, and reporting cyberattacks.

### Results of Syntheses

A meta-analysis was not conducted because effect sizes were not reported.

### Prevention, Detection, Response, Recovery, and Reporting Cyberattacks

Table 2 summarizes the techniques associated with prevention, detection, response, recovery, and reporting cyberattacks in hospitals. These are organized into 12 themes and one observation.

**Table 2:** Prevention, detection, response, recovery, and reporting of cyberattacks

Prevention, detection, and reporting themes	Reference	Frequency
Organizational cyber resiliency	21 23 25 27 28 30 31 32 35 36 37 38 39 40 41 a	41
Monitoring tools and technology	22 23 24 27 35 37 41 a	20
Standard maintenance and practice	23 25 28 31 35 37 41 a	13
Encryption and DLP	26 28 29 30 31 34 36 41 a	13
Risk-based management of cybersecurity	27 37 38 41 42 a	11
AI, AIX, or active AI	30 34 36 a	9
Blockchain	30 33 34 38 a	8
Access control	21 26 34 35 36 37 41 a	8
Segmented network	21 28 33 35 36 37 41	7
Governance and leadership	21 27 31 39	4
Disable legacy protocols and equipment that cannot be updated	35 36	2
Postevent analysis	31 39	2
Big data	34	1

a More than one observation recorded in articles.

Organizational cyber resilience appeared in 41 of 139 observations (30%). 21 23 25 27 28 30 31 32 35 36 37 38 39 40 41 This theme encompassed an array of observations: educating staff on passwords, 21 halt all elective surgeries and invasive procedures, 23 education and training, 25 27 28 31 32 35 37 38 40 41 culture of awareness and personal responsibility, 25 cybersecurity practices integrated with normal workload, 25 organizational cyber resiliency, 27 protection at the physical, network, and application layers, 30 cyberattack plan, 31 establish and practice procedures, 35 39 41 continuity of operations, 31 35 third-party penetration testing, 35 regular backups, 35 36 forensic analysis of affected devices, 35 secure IoT devices, 36 intelligence gathering capabilities, perform recovery operations if the electronic health record (EHR) is held for ransom, 36 prevention with passive network monitoring (PNM), use PNM to protect without disrupting function of IoMT, medical device cybersecurity program, application security, security information and event monitoring, 37 cybersecurity insurance that covers ransomware attacks and third-party contractors, capabilities assessment, 38 establish multi-level decision making. 39

The standard maintenance and practices taught to all network managers and security professionals appeared in 13 of 139 observations (9%). This theme included those practices not captured by any cyber resilience program such as distribution of laptops with secured cellular Internet connection, 23 update the IT infrastructure, 25 software patching, 28 35 virtual private networks, regular policy and procedure review, 31 disconnect systems that cannot be updated, test backups, store backups offsite, 35 secure cloud-based applications, 36 regular maintenance, practice integrity in preventing waste, fraud, and abuse, 41 and general system protection.

Encryption and data loss prevention (DLP) occurred in 13 of 139 observations (9%). This theme included elliptic curve cryptography (ECC) based key management and Diffie–Hellman key exchange, 26 28 41 alternate quantum random walks, controlled Rubik's Cube transformations, and ECC with Hill Cipher (ECCHC), 29 asymmetric key cryptography, 30 attribute-based encryption, encryption of immutable storage, 34 and DLP. 36

Risk-based management of cybersecurity appeared in 11 of 139 observations (8%). This theme included cyber-risk frameworks, 27 41 identify mission-critical assets using the NIST Cybersecurity Framework, overview of network security types, identify programmatic gaps that signal the need for PNM, mobile device security, 37 perform a comprehensive assessment of IT capabilities before purchasing a policy, 38 develop enterprise risk management, and establish a risk-management culture. 42

The theme AI, AIX, or active AI appeared in 9 of 139 observations (6%). This theme included neighborhood component analysis, 22 machine learning to identify cyberattacks in the IoHT, 22 24 30 efficient feature engineering, explainability through Shapley Additive Explanation, 24 real-time AI to interrogate packets, 30 and record real-time success or failure of user activities. 36 Researchers noted the importance of using a combination of these practices as they serve different purposes.

Two themes appeared in 8 of 139 observations (6%): blockchain and access control. The blockchain theme included private blockchain-based federated learning, a Linux-based hyperledger, 30 and a wide range of applications of blockchain to secure the electronic medical record (EMR), 33 34 38 remote patient monitoring, and health insurance claims. 33 The access control theme included multi-factor authentication, 21 35 36 37 41 authentication, authorization, and access (AAA) control layer, 26 access control through blockchain, 34 role-based security, 35 and a zero standing privilege environment. 36

Segmenting the network appeared in 7 of 139 observations (5%). This theme included segmenting networks to reduce the amount of data or systems one person or system can access 21 28 33 35 36 37 and segregate billing functions. 41

Governance and leadership appeared in 4 of 139 observations (3%). This theme included observations such as a decision to resort to paper records and notes as a backup, 21 create a communications plan, and establish strong horizontal and vertical leadership. 27 31 39

Two themes appeared in 2 of the 139 observations (1%): disabling legacy protocols and equipment, and postevent analysis. The disabling legacy protocols and equipment that cannot be updated theme included the removal of the remote desktop protocol (RDP) and secure shell (SSH) for remote desktop. 35 36 The postevent analysis theme included analyzing affected systems after the cyberattack. 31 39 Finally, Big Data appeared once in 139 observations (1%). 34

### Additional Analysis Performed

#### Effects on Patient Care

Three themes across eleven observations were identified in the literature as related to patient care. Six of 11 observations (55%) identified a delay in care. 21 23 33 36 These included disruptions of workflow, rescheduling surgeries and nonlife threatening procedures, patient transfers, excess network traffic, and reverting to paper records. Three of 11 observations (27%) identified potential medical error. 28 36 37 These included how easily infusion pumps can be altered to change rates of flow, how reverting to paper records causes manual medication dispensing, and how anti-virus software can disrupt the functioning of IoMT. Two of 11 observations (18%) identified missing care elements such as lab results or images. 21

## Discussion

### Summary of Evidence

Through a filtered search from four research databases, reviewers identified 139 observations that followed along 13 themes: organizational cyber resiliency, 21 23 25 27 28 30 31 32 35 36 37 38 39 40 41 monitoring tools and technology, 22 23 24 27 35 37 41 standard maintenance and practice, 23 25 28 31 35 36 37 41 encryption and DLP, 26 28 29 30 31 34 35 36 41 risk-based management of cybersecurity, 27 37 38 41 42 AI, AIX, or active AI, 22 24 30 34 36 blockchain, 30 33 34 38 access control, 21 26 34 35 36 37 41 segmented network, 21 28 33 35 36 37 41 governance and leadership, 21 27 31 39 disabled legacy protocols and equipment that cannot be updated, 35 36 postevent analysis, 31 39 and big data. 34 These findings are commensurate with other research in this area. 7 9 10 11 The elements of the ECHO model track closely with those in this review: both identified governance, strategy, awareness, risk management, technical capabilities, training, and education. 7 The challenges identified by Joshi were not all detected in this systematic review, but data security was a common element. 9 All but one of the weaknesses identified by Salama et al were found in this review: security awareness, endpoint device management, continuity of operations planning, coordinated incident response—the one not covered was spending on security versus service delivery. 10 Finally, the technical measures identified by Dobrovolska et al were found in organizational cyber resilience, but the legal measures were not identified by this research. 11

This work highlights many of the key elements of cyber resilience programs. Two programs in the United States include most of what was found in this review. The company SSL2Buy offers a cyber resilience model that includes regular monitoring, report generation, security protocols, regular maintenance, access control, data protection, data recovery, training, backup of data, and blocking. 43 Another model by Black Swan Security includes security initiative and problem solving, pace of decision making, diversity of cyber capacity, organizational readiness and business problem solving, technical agility and adaptation, and situational awareness. 44 Finally, the United States lags behind the European Union in cybersecurity legislation. In 2024, the EU passed the Cyber Resilience Act, which mandates cybersecurity requirements for all vertical levels of software development and sales. 45 The EU also covers medical devices separately. This cyber resilience initiative bears a strong resemblance to the U.S. Office of the National Coordinator for Health Information Technology's (ONCHIT) program promoting interoperability of EMRs and security of health information from 2004. 46 Organizations with a robust program in cyber resilience will address many of the practices identified in this review.

One of the new items in this review was the use of blockchain to secure data. To be clear, this was blockchain on a private network, not to be confused with public blockchain, whose “proof of work” can consume electricity on the same scale as the country of Taiwan. One quasi-experimental study used a federated learning model coupled with asymmetric key cryptography (encryption) and a Linux-based hyperledger (private blockchain) to protect data at the physical, network, and application layers of the OSI model and machine learning (AI) to identify cyberattacks in the IoHT. 30 47 Other studies used blockchain to secure several aspects of the healthcare cash cycle, such as the EMR, remote patient monitoring, and health insurance claims. 33 34 38

The last unique items in this review revolved around AI, AIX, and active AI. The combination of these powerful tools strengthened defenses, enabled faster detection, and enabled a more coordinated response. 22 24 30 34 36 These articles highlighted how medical devices, mostly summarized with the acronym IoMT, have greatly enhanced the quality of life of patients and ease of data transfer to providers; they also pose a threat to both the hospital network and to the patient's safety if they are breached. AI tools like efficient feature engineering and explainability through Shapley Additive Explanation provide some protection to both the organization and the patient.

### Practical Implications

For the healthcare providers and administrators, this research highlights the importance of ensuring the cyber resilience of the organization and ensuring the IT staff are current in training and practices. The IT staff should creatively seek new methods of securing patient data, detecting intrusions, responding, and reporting appropriately. IT security is an expensive operation, but the alternatives are unacceptable.

While some practitioners would prefer to outsource cybersecurity, the literature suggests otherwise. Third-party actors often do not conform to the same governance procedures or policies for identity lifecycles, which greatly complicates access management. 48 Because third-party data breaches are on the rise, in-house cyber resilience is a top priority.

It is important to recognize the patient's impact on some of the themes. For instance, patient-centered outcomes include delays in care, diversion, and adverse events. Care-delivery outcomes include length of stay, emergency department throughput, door-to-provider, and operating-room disruption. Financial outcomes include the financial cost to organizations that ignore the themes identified. Feasibility outcomes include the training burden and opportunity cost. Resiliency outcomes include integrity restoration time and data-loss recovery.

### Future Research

While the results of this research provide new tools not highlighted in other reviews, it is important that methodologically rigorous research be performed in cybersecurity to protect the healthcare infrastructure. Future research should use blockchain technology, in combination with AI, AIX, and active AI, to compare the prevention, detection, response, recovery, and reporting of cyberattacks with defenses that do not include these tools. Additionally, policymakers should help codify blockchain standards in healthcare legislation to ensure a robust defense. Future research should also bridge the divide created by cyber threats and their associated effects on patient care.

### Limitations

A major limitation of this review is its lack of RCTs or nonrandomized study interventions (NRSI) in the area of cybersecurity. This review consisted of sixteen white papers: one quasi-experimental, one qualitative, one observational study, one mixed-methods, one nonexperimental, and one true experiment. Another limitation is the limit of Boolean search terms to MeSH. It is possible that other key terms to this topic may have been found outside of MeSH terms.

## Conclusion

Techniques to prevent, detect, respond, and report cyberattacks largely surround proper organizational cyber resiliency. But they are also augmented by monitoring tools, standard IT maintenance and practice, encryption and DLP, risk-based management, AI, AIX, and active AI, network segmentation, governance and leadership, disabling legacy protocols and systems that cannot be updated, postevent analysis, and big data. These tools compel IT professionals to remain current in their IT skills and to remain both creative and flexible in the face of cyberattacks. While practices like segmentation require close coordination between clinicians, administrators, and IT professionals, the threats are too great to ignore. Such practices can be managed at the institutional level.

## Clinical Relevance Statement

Providers and administrators should be aware of the implications for their security posture and cyber practices. Providers and administrators should ensure that measures are taken to protect the network, the infrastructure, and patient data. Cyber resiliency, monitoring tools, standard IT maintenance and practice, encryption and DLP, risk-based management, AI, AIX, and active AI, network segmentation, governance and leadership, disabling legacy protocols and systems that cannot be updated, postevent analysis, and big data are strongly recommended cybersecurity tools for the clinical workspace.

## Multiple-Choice Questions

1. What AI tools are available to enhance cyber resilience?
  - Standard AI
  - AIX
  - Active AI
  - All of the above
  **Correct Answer** : The correct answer is option d. All of the above. All three AI tools are available to enhance cyber resilience, and they are each very important.
2. What basic security practices should be omnipresent?
  - Encryption
  - Disabling systems that can no longer be updated.
  - Network segmentation
  - All of the above
  **Correct Answer** : The correct answer is option d. All of the above. The practices are taught to all information technology professionals in for education such as computer science, in professional certifications such as CompTIA Security+, and in organizational training, particularly in the sector of healthcare.
